# Supplementary material for: FlexENN: A Graph Neural Network for Binding Energy Prediction of Globular and Intrinsically Disordered Proteins
Source: J Chem Inf Model. 2026 Oct 9;66(21):13795–808. doi: 10.1021/acs.jcim.6c01545 (PMC13753731; doi:10.1021/acs.jcim.6c01545)
Supplement: Supplementary file 1 [file ci6c01545_si_001.pdf]

Supporting Information for FlexENN: A Graph Neural Network for Binding Energy Prediction of Globular and Intrinsically Disordered Proteins

Maryum Irshad[a], Kassandra Ori-McKenney[b], Ruxandra I. Dima[a]

[a]Department of Chemistry, University of Cincinnati, Cincinnati, Ohio, 45221, United States

[b]Department of Molecular and Cellular Biology, University of California Davis, Davis, California, 95616, United States

Evaluation on MD-derived Structural Ensembles

To assess the robustness of FlexENN-F to conformational variability beyond a single experimentally resolved structure, the prediction model was additionally tested on structures sampled from all-atom molecular dynamics (MD) simulations of the CDC-MT complex. Five frames were sampled from the final 100 ns of each of five independent trajectories, yielding a total of 25 equilibrated structures. FlexENN-F predicted an average binding energy value of -7.95 ± 0.82 kcal/mol across these frames, demonstrating relatively consistent predictions across independently sampled conformations. This analysis was restricted to CDC because the graph representations generated from MD snapshots remained within the range of edge counts represented in the FlexENN-F training distribution (Figure S5). Notably, the simulated CDC-MT complex also contains the tubulin C-terminal tails (CTTs), whereas the experimentally resolved CDC structure used in the primary analysis contains only the resolved CDC-MT interface, providing a structurally more complex test of the model. Other MD-derived systems generated substantially larger interface graphs as conformational sampling introduced additional residue contacts, placing their edge counts outside the range adequately represented during training. Thus, these results indicate that FlexENN-F can provide stable predictions across multiple conformations when the resulting interface graphs remain within its learned structural domain, while also highlighting the current limitation imposed by the limited representation of larger, more highly connected interfaces in the training dataset.

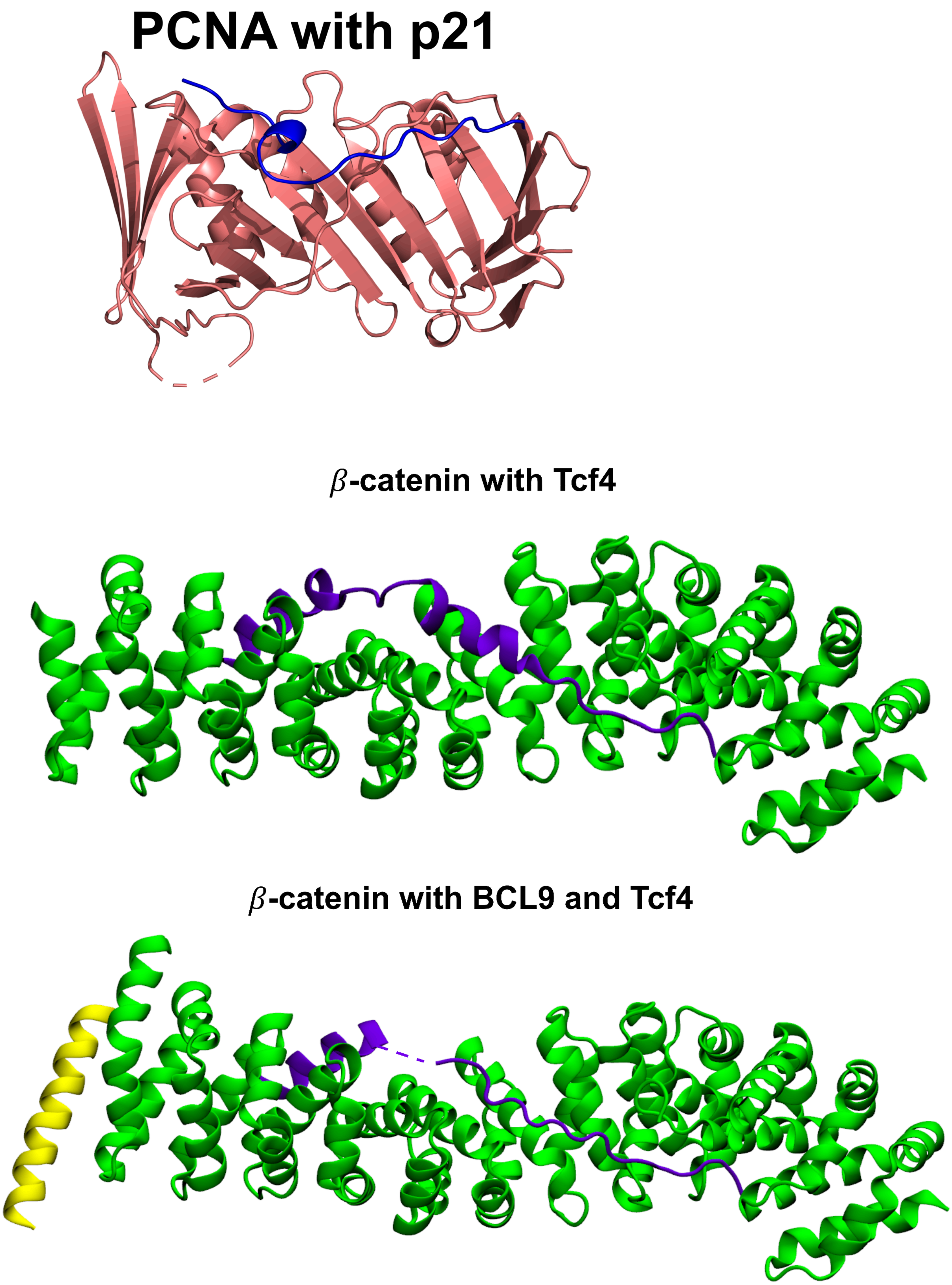

Figure S1. PDB structures of the second benchmark subset of IDPs.

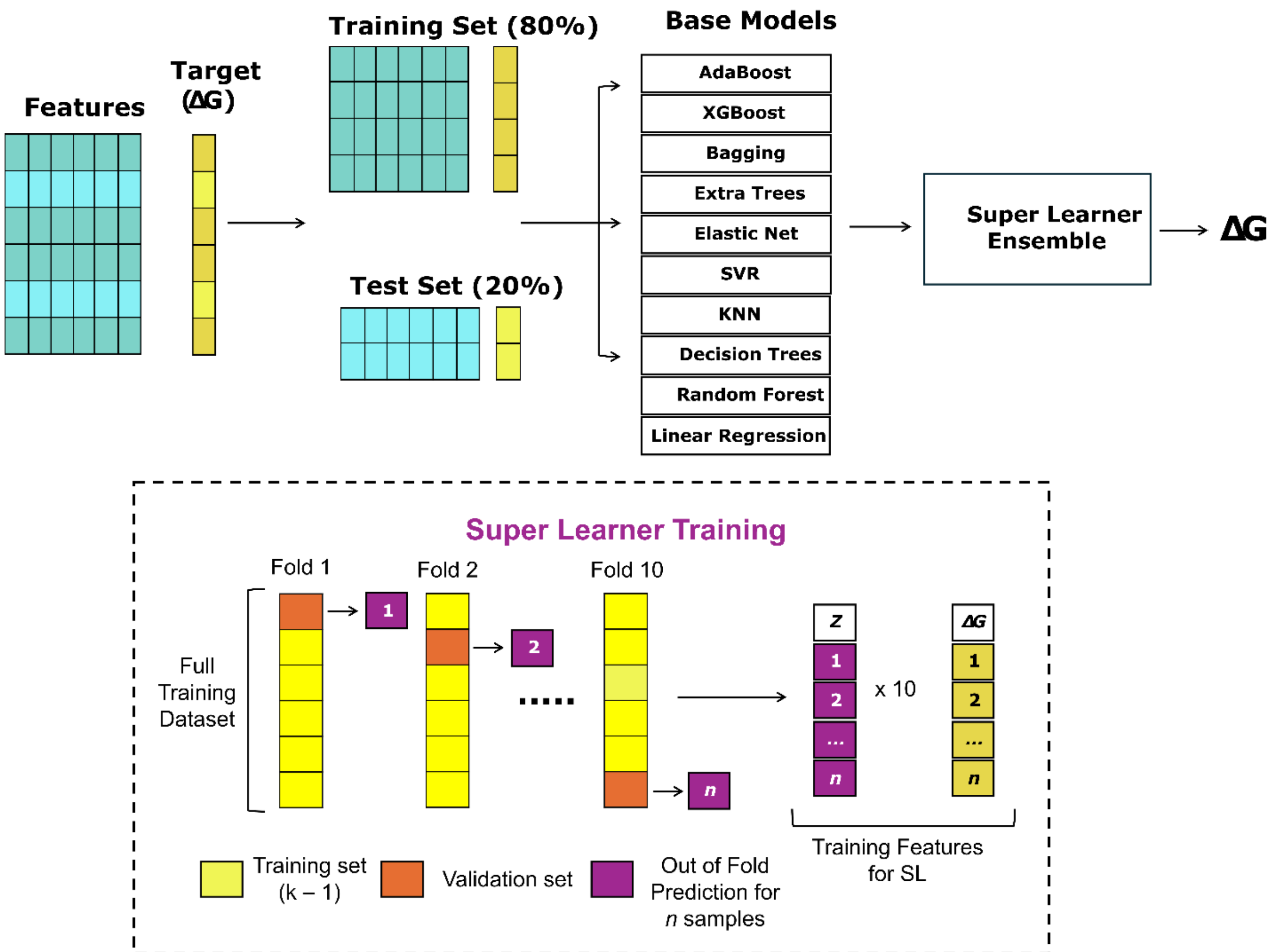

Figure S2. Schematic representation of the SL algorithm adapted from PBEE. The full training set is used to train 10 different ML algorithms. For the SL training, the dataset was split into 10-folds, and the subsets were trained on each ML algorithm and evaluated using k-fold cross-validation. All out-of-fold predictions were stored as a covariance matrix, and the model was fit to the subset and stored. The SL model was generated by combining predictions from each candidate using a weighted linear regression of their outputs on the full dataset.

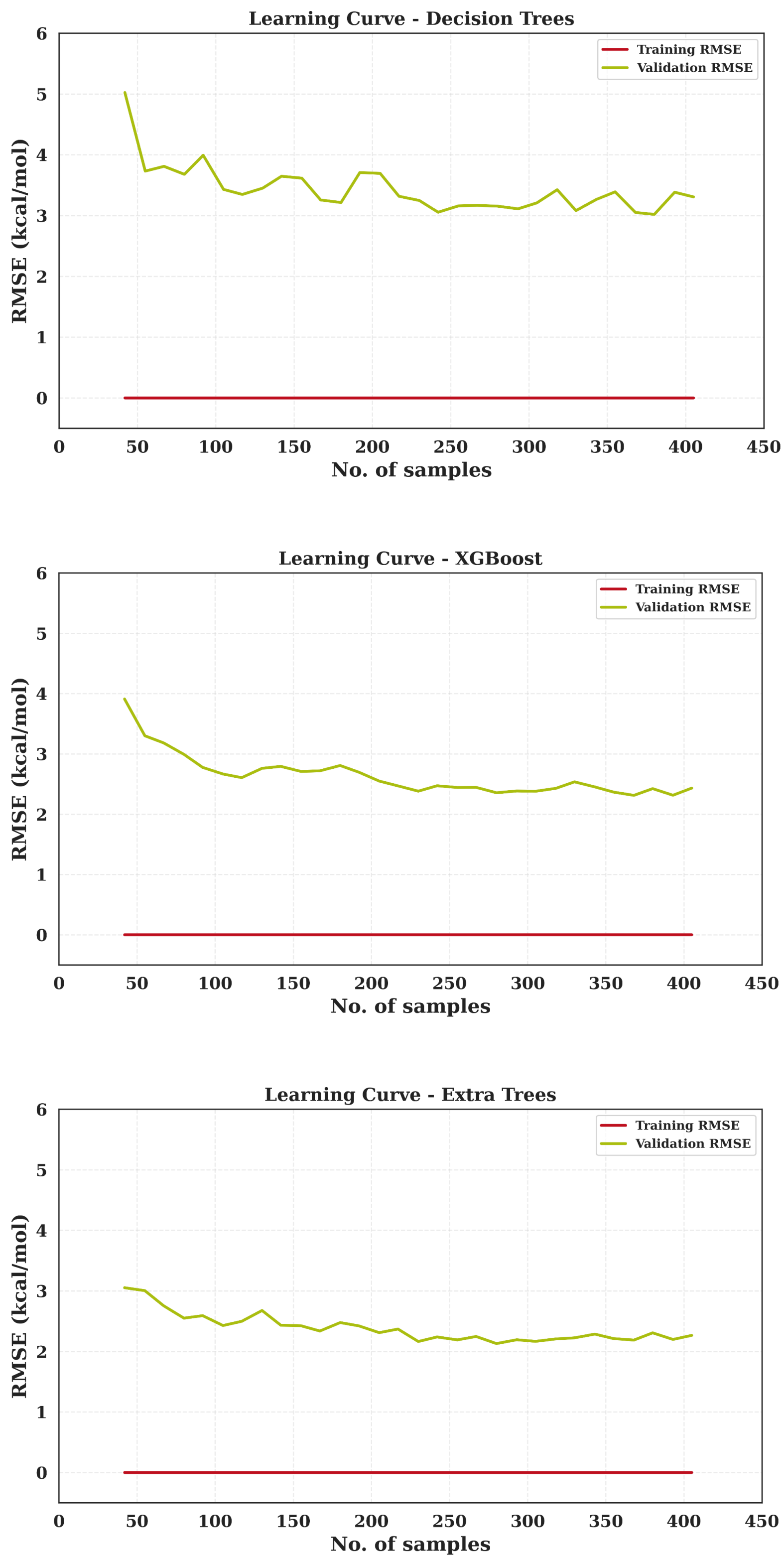

Figure S3. Learning curves for Decision Trees, XGBoost, and Extra Trees show clear overfitting, as evidenced by zero training error on the original PBEE Rosetta feature set.

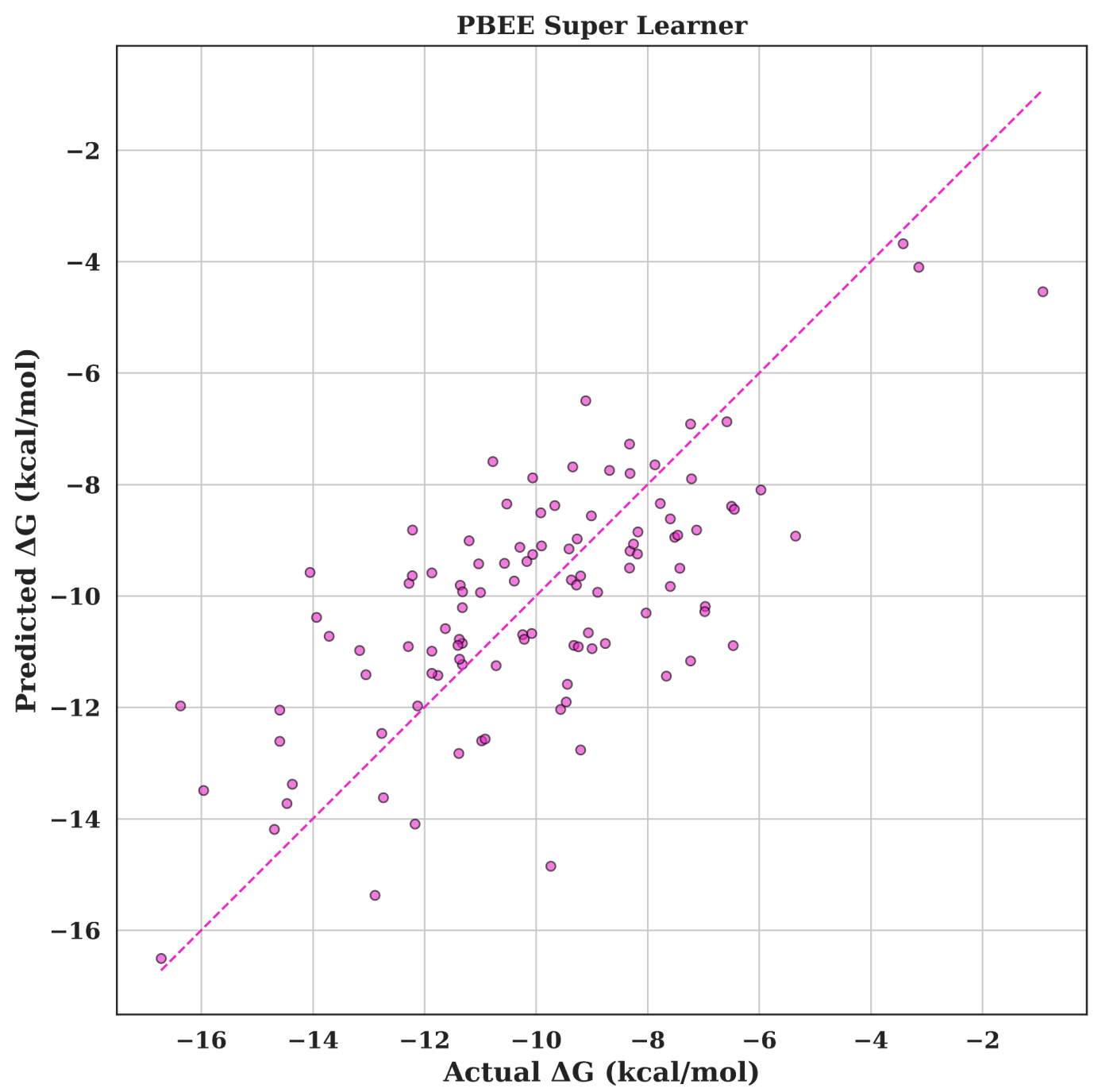

Figure S4. True versus predicted values generated from the Super Learner trained on the original PBEE dataset.

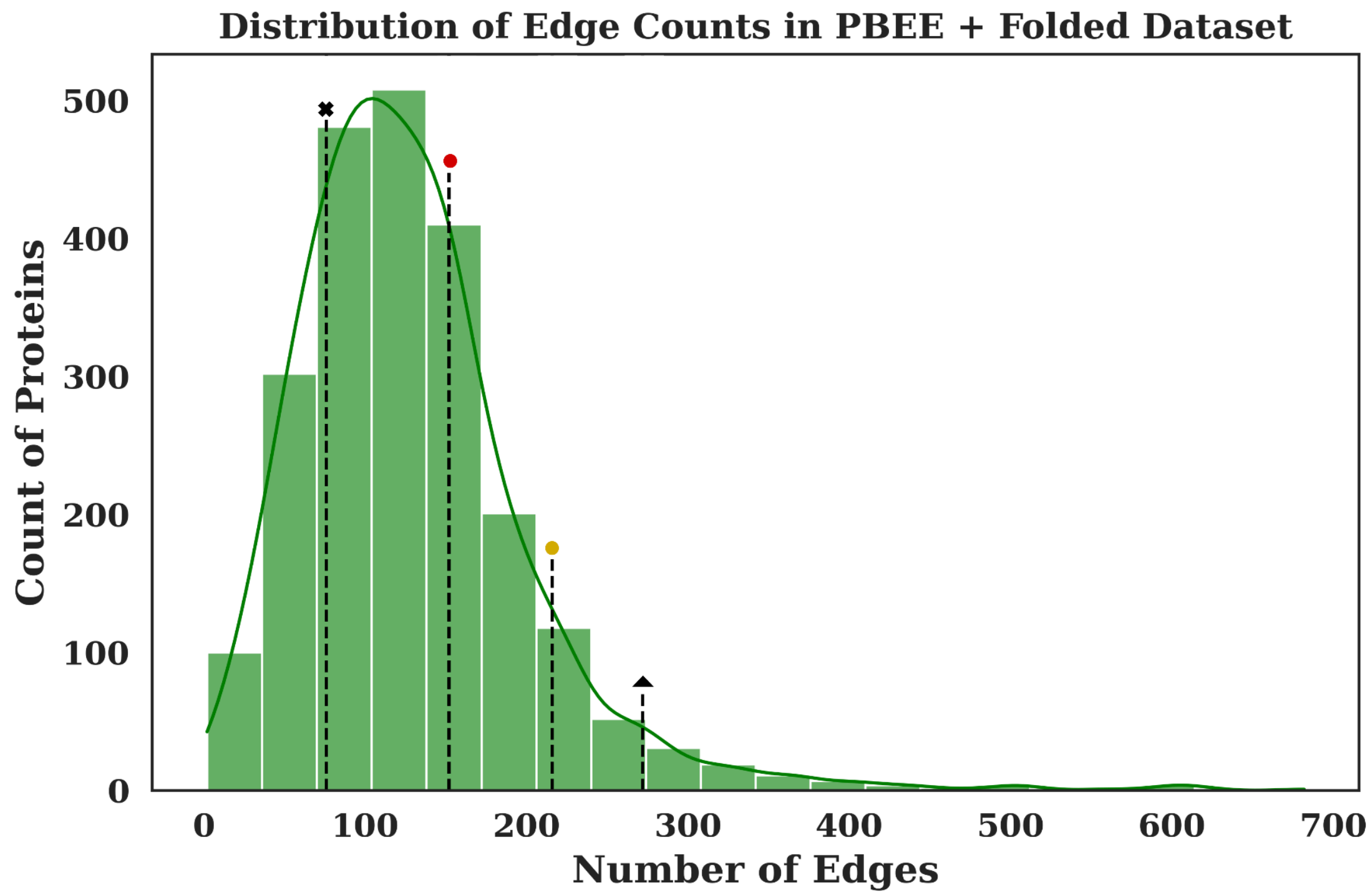

Figure S5. Distribution of edge counts per protein in the PBEE + Folded dataset. Vertical dashed lines indicate edge counts for reference benchmark folded MT-MAP structures: Tubulin lateral interface (✖, 76), CDC-MT (●, 152), NDC-MT (●, 216), and Tubulin interdimer/intradimer interface (▲, 272).

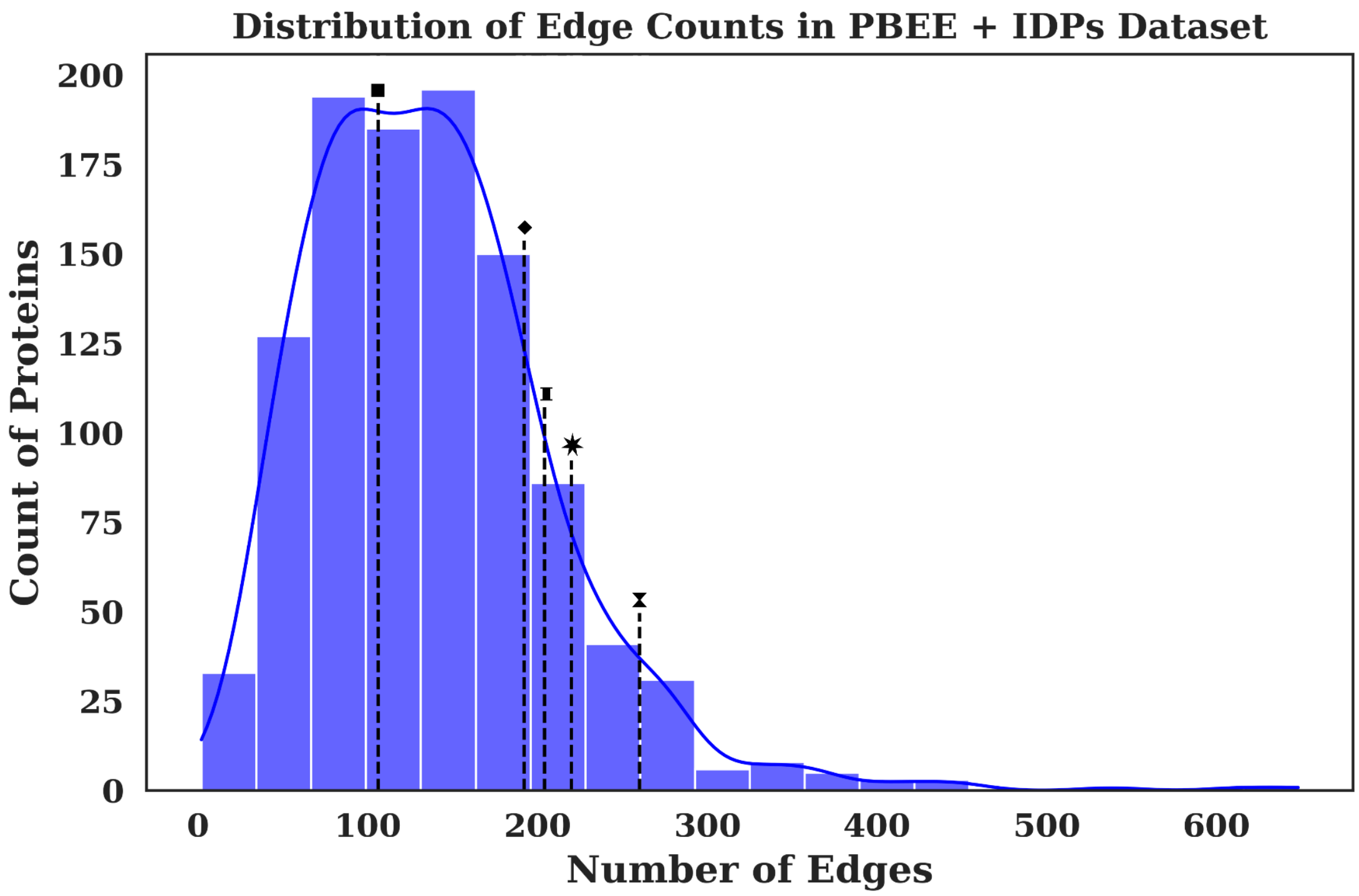

Figure S6. Distribution of edge counts per protein in the PBEE + IDPs dataset. Vertical dashed lines indicate edge counts for reference benchmark IDPs structures: MAP7-MT (■, 106), Tau-MT (◆, 192), β-catenin-Tcf4 (**I**, 204), PCNA-p21 (✶, 220), and β-catenin-Tcf4/BCL9 (⧗, 260)

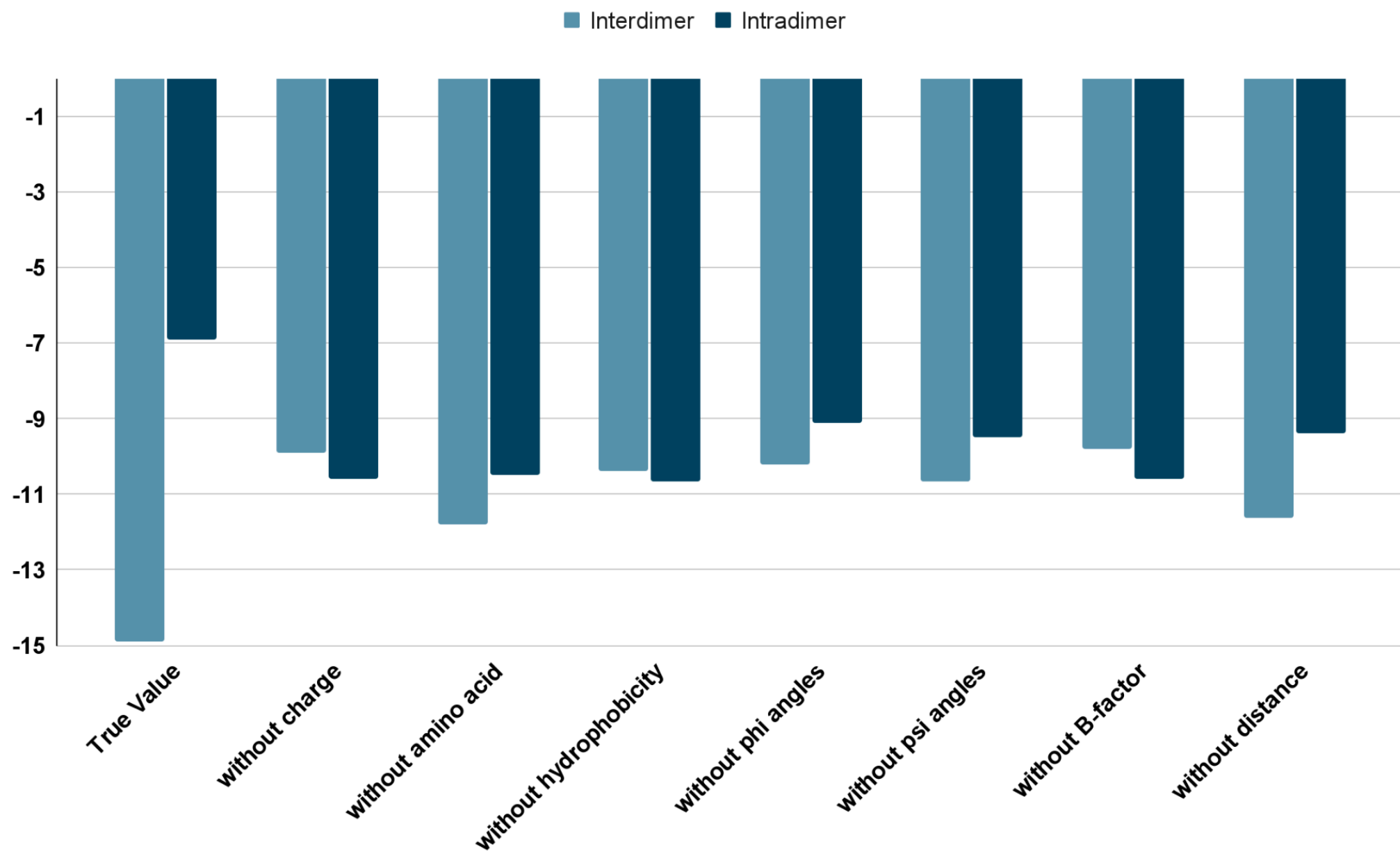

Figure S7. Ablation analysis of the FlexENN-F architecture for the prediction of ΔG values for MT's interdimer and lateral interface

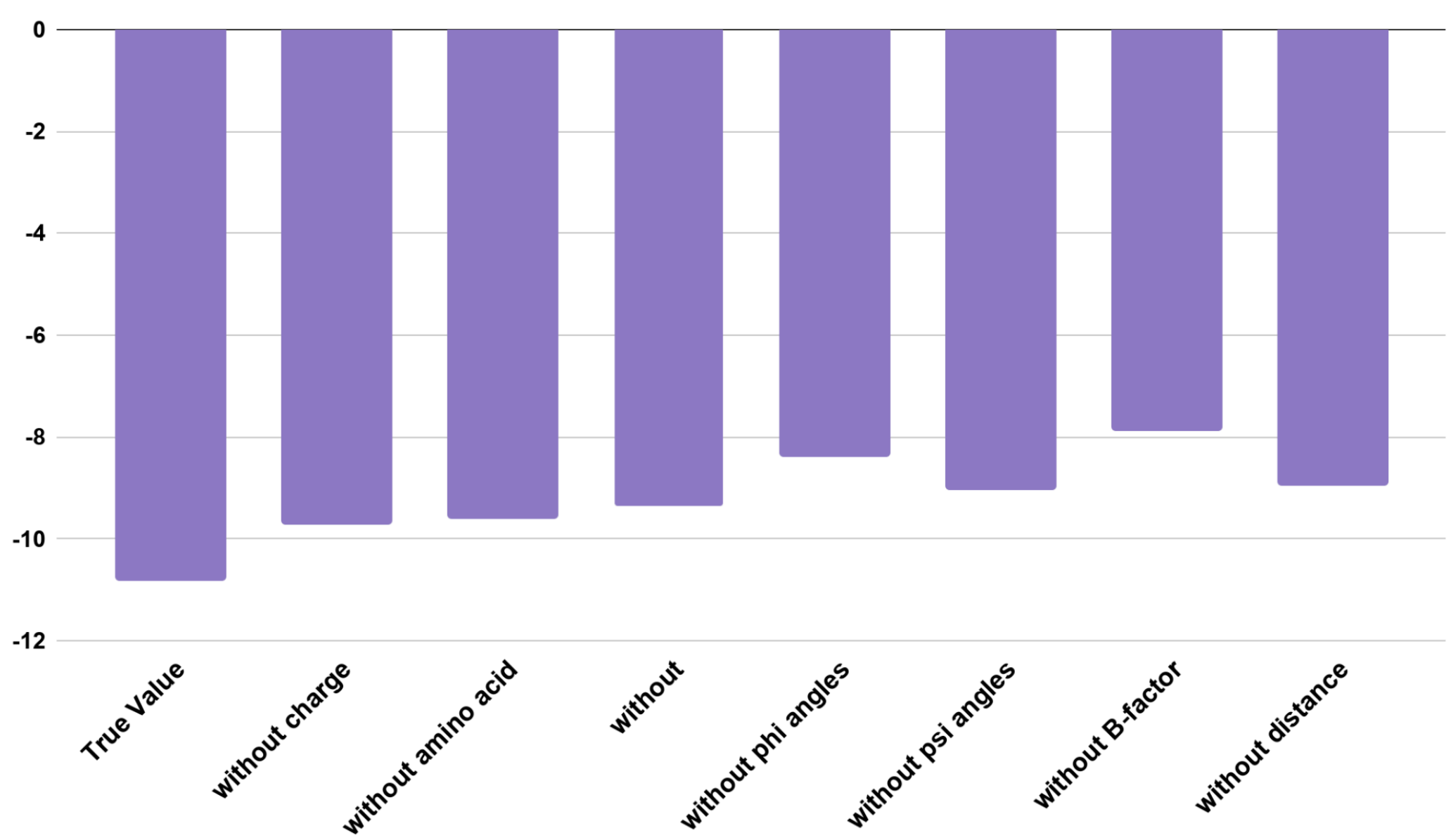

Figure S8. Ablation analysis of the FlexENN-F architecture for the prediction of ΔG values for DCX

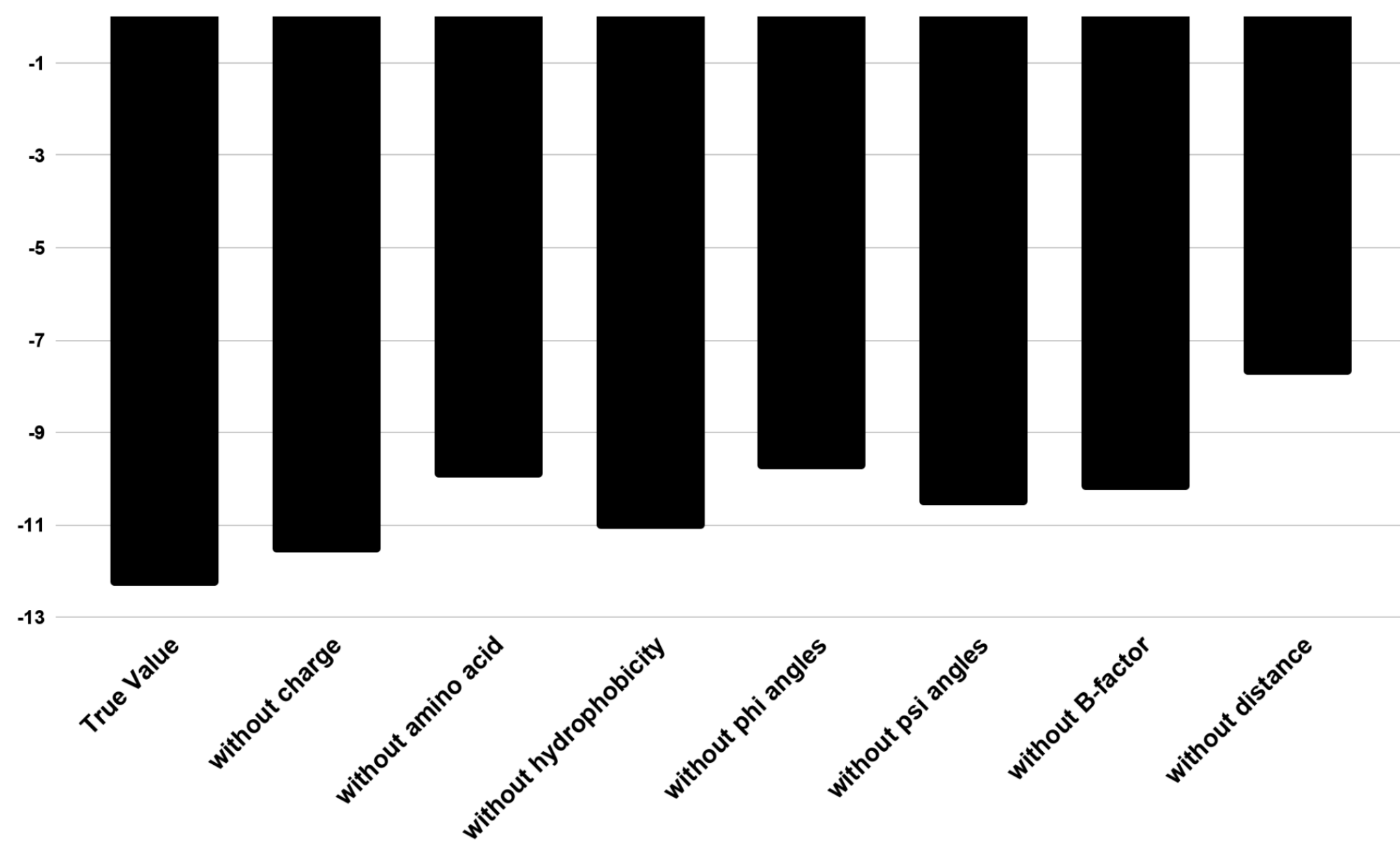

Figure S9. Ablation analysis of the FlexENN-I architecture for the prediction of ΔG values for MAP7
